# Prior Physical Performance Impacts Social Contact and Social Participation of U.S. Older People During COVID-19

**DOI:** 10.1093/geroni/igab046.1776

**Published:** 2021-12-17

**Authors:** Yun Zhang, Wei Hou, Sean Clouston

**Affiliations:** 1 Renaissance School of Medicine, Stony Brook University, Stony Brook, New York, United States; 2 Renaissance School of Medicine, Stony Brook University, Renaissance School of Medicine, Stony Brook University, New York, United States

## Abstract

**Objective:**

To assess what roles the prior physical and cognitive performances of US older people played in changes of social contact and social participation due to the COVID-19 pandemic.

**Methods:**

We used the 2020 National Health and Aging Trends Study (NHATS) COVID-19 questionnaire (n=3,188, response rate=80.5%), linked to demographic information from the 2019 NHATS wave. We excluded participants who reported COVID-19 diagnosis and/or symptoms (n=288), and those represented by a proxy (n=414). We compared older people’s social contact with family and friends, social contact with health care providers, and social participation, prior to and during the COVID-19. We used paired t-test for the summed scores, Wilcoxon signed-rank for paired categorical variables, and McNamara test for paired binary variables. We further used weighted linear and logistic regressions adjusted for multiple covariates to investigate the effects of prior physical and cognitive performances on social contact and social participation, prior to, during, and changes in the COVID-19.

**Results:**

The sample included 2,486 participants that were predominantly females (56.20%) and non-Hispanic whites (88.43%), with participants averaging 78.24 years old. The COVID-19 pandemic resulted in significantly increased social contact with family and friends but decreased social participation of the US older people. Better prior physical performance was associated with significantly increased video calls and volunteering work during the COVID-19, while prior cognitive ability was not a significant risk factor.

**Conclusion:**

Prior physical performance, rather than prior cognitive ability, significantly affected the social way old people responded to a pandemic crisis.

